# The *Lactococcus lactis* Pan-Plasmidome

**DOI:** 10.3389/fmicb.2019.00707

**Published:** 2019-04-04

**Authors:** Philip Kelleher, Jennifer Mahony, Francesca Bottacini, Gabriele A. Lugli, Marco Ventura, Douwe van Sinderen

**Affiliations:** ^1^School of Microbiology, University College Cork, Cork, Ireland; ^2^APC Microbiome Ireland, University College Cork, Cork, Ireland; ^3^Laboratory of Probiogenomics, Department of Chemistry, Life Sciences and Environmental Sustainability, University of Parma, Parma, Italy

**Keywords:** lactococcal, plasmid, SMRT sequencing, dairy fermentation, conjugation, lactic acid bacteria

## Abstract

Plasmids are autonomous, self-replicating, extrachromosomal genetic elements that are typically not essential for growth of their host. They may encode metabolic capabilities, which promote the maintenance of these genetic elements, and may allow adaption to specific ecological niches and consequently enhance survival. Genome sequencing of 16 *Lactococcus lactis* strains revealed the presence of 83 plasmids, including two megaplasmids. The limitations of Pacific Biosciences SMRT sequencing in detecting the total plasmid complement of lactococcal strains is examined, while a combined Illumina/SMRT sequencing approach is proposed to combat these issues. Comparative genome analysis of these plasmid sequences combined with other publicly available plasmid sequence data allowed the definition of the lactococcal plasmidome, and facilitated an investigation into (bio) technologically important plasmid-encoded traits such as conjugation, bacteriocin production, exopolysaccharide (EPS) production, and (bacterio) phage resistance.

## Introduction

*Lactococcus lactis* is globally applied as a starter culture for dairy-based food fermentations, such as those involved in the production of Cheddar, Colby, Gouda and blue cheeses, and from an economic and (food) biotechnological perspective represents one of the most important bacteria (Ainsworth et al., 2014a). It is widely accepted that *L*. *lactis* originated from a plant-associated niche (Price et al., 2012; Wels et al., 2019) and, whilst the majority of sequenced lactococcal representatives are isolated from the dairy environment, this is not representative of the presumed diversity of the taxon. It is evident from genome analyses of *L. lactis* strains isolated from the dairy niche that genome decay (due to functional redundancy) (Makarova et al., 2006; Goh et al., 2011; Ainsworth et al., 2013; Kelleher et al., 2015, 2017), in parallel with the acquisition of novel plasmid-encoded traits played a significant role in their adaptation to the nutrient-rich environment of milk. Analysis of the plasmid complement has revealed a relatively low abundance of plasmids among lactococcal strains isolated from non-dairy niches (Makarova et al., 2006; Kelly et al., 2010; Ainsworth et al., 2013, 2014c). Since various dairy-associated phenotypes are encoded by plasmids, horizontal acquisition to adapt to the dairy environment is likely to be one of the major drivers of plasmid transfer in *L. lactis* (Ainsworth et al., 2014c) with dairy strains containing up to twelve plasmids (Van Mastrigt et al., 2018a). Plasmid transfer in *L. lactis* is believed to be predominantly governed by conjugation and transduction (Ainsworth et al., 2014c), but may also occur as a result of transformation (David et al., 2017; Mulder et al., 2017) Transduction is a process in which DNA transfer is carried out by a (bacterio)phage (i.e., a virus that infects a bacterium) due to unintentional packaging of host DNA, and has previously been observed in *L*. *lactis* (Ammann et al., 2008; Wegmann et al., 2012). Conjugation involves the transfer of plasmid material via a conjugative apparatus (Grohmann et al., 2003) and is of particular importance as it represents a natural phenomenon that is suitable for the transfer of genetic traits such as phage resistance systems in food grade processes, bacteriocin production (including nisin), proteinases, and citrate utilization (Neve et al., 1987; Kojic et al., 2005; Mills et al., 2006; Van Mastrigt et al., 2018b). Extensive research into the technological traits of *L*. *lactis* has been carried out in the past with a significant focus on lactose utilization (Van Rooijen and De Vos, 1990; Van Rooijen et al., 1992), casein metabolism (Siezen et al., 2005), citrate metabolism (Drider et al., 2004; Van Mastrigt et al., 2018a), flavor formation (McSweeney and Sousa, 2000; McSweeney, 2004), and phage resistance mechanisms (Labrie et al., 2010), all of which represent properties that are commonly plasmid-encoded. Lactose utilization in *L*. *lactis* is governed by the *lac* operon, which provides dairy strains with the ability to rapidly ferment lactose and grow in milk. The *L. lactis lac* operon, which consists of the genes *lacABCDEFGX*, is generally plasmid-borne and is regulated by a repressor, encoded by the adjacent *lacR* gene (Van Rooijen and De Vos, 1990; Van Rooijen et al., 1992). Citrate metabolism is conducted by citrate-positive (Cit^+^) lactococci and is important as it leads to the production of a number of volatile flavor compounds (McSweeney and Sousa, 2000). Citrate uptake and subsequent diacetyl production is governed by the plasmid-encoded *citQRP* operon in lactococcal species (Drider et al., 2004). Proteolysis also significantly contributes to flavor production in fermented dairy products, although high levels of proteolysis may cause bitterness in cheese (Broadbent et al., 2002). The plasmid-encoded extracellular cell wall proteinase (lactocepin) has been shown to be directly associated with the bitter flavor defect in Cheddar cheese varieties, specifically involving starters which produce lactocepin of the so-called a, e, or h groups, and its characterization is of particular importance when selecting novel starter cultures (Broadbent et al., 2002).

Lactococcal phages are recognized as the main cause of fermentation problems within the dairy industry with concomitant economic problems. Lactococcal strains possess an arsenal of phage defense mechanisms, such as R-M systems and abortive infection (Abi) systems, many of which are plasmid-encoded. In the current study, we assess the genetic content of lactococcal plasmids, define the current pan-plasmidome of *L*. *lactis*, and investigate plasmid-encoded (and technologically relevant) traits.

## Materials and Methods

### Sequencing

In total, 83 plasmids (81 plasmids and 2 megaplasmids, the latter defined as plasmids that are >100 Kbp in length) were sequenced in the context of this study (Table 1). Sequencing of sixteen lactococcal strains was performed as previously described (Kelleher et al., 2017) utilizing the SMRT sequencing approach on a Pacific Biosciences RS II sequencing platform (executed by GATC Biotech Ltd., Germany). *De novo* assemblies were performed on the Pacific Biosciences SMRTPortal analysis platform (version 2.3.1), utilizing the RS_HGAP_Assembly.2 protocol. Assemblies were then repeated with a reduced minimum coverage threshold adjusted to 15X to ensure all plasmid-associated contigs had been detected.

**Table 1:** Characteristics of the plasmids analyzed in this study.

Name	Accession	Size (Kbp)	GC (%)	Genes	Niche	Replication mode
KLDS 4.0325 p1	CP006767	4.094	30.02	4	Fermented food	RCR
KLDS 4.0325 p2	CP007042	0.870	32.64	2	Fermented food	Undetermined
KLDS 4.0325 p3	CP007043	1.278	32.63	4	Fermented food	Undetermined
KLDS 4.0325 p4	CP029291	9.000	31.02	11	Fermented food	Theta
KLDS 4.0325 p5	CP029292	47.268	34.43	41	Fermented food	Theta
KLDS 4.0325 p6	CP029293	109.112	35.38	90	Fermented food	Theta
p14B4	CP028161	59.700	33.69	58	Plant	Theta
p158A^∗^	CP016685	75.119	33.04	93	Dairy	Theta
p158B^∗^	CP016686	57.981	33.56	22	Dairy	Theta
p158C^∗^	CP016687	51.651	34.57	55	Dairy	Theta
p158D^∗^	CP016688	33.287	37.39	32	Dairy	Theta
p158E^∗^	CP016689	11.679	34.05	13	Dairy	Theta
p158F^∗^	CP016690	6.164	35.84	4	Dairy	Theta
p158G^$^	CP034596	2.064	33.38	3	Dairy	RCR
p184A^∗^	CP016691	9.735	34.84	13	Dairy	Theta
p184B^∗^	CP016692	5.929	34.51	6	Dairy	Theta
p184C^∗^	CP016693	10.488	33.35	14	Dairy	Theta
p184D^$^	CP034584	2.052	30.64	3	Dairy	RCR
p184E^$^	CP034585	5.900	33.85	4	Dairy	Theta
p184F^$^	CP034586	8.312	34.74	8	Dairy	Theta
p229A^∗^	CP016694	56.368	34.81	59	Dairy	Theta
p229B^∗^	CP016695	33.280	37.39	29	Dairy	Theta
p229C^∗^	CP016696	30.272	35.15	29	Dairy	Theta
p229D^∗^	CP016697	6.153	35.88	8	Dairy	Theta
p229E^∗^	CP016698	39.612	32.40	51	Dairy	Theta
p275A^∗^	CP016699	92.710	35.35	104	Dairy	Theta
p275B^∗^	CP016700	56.332	33.36	65	Dairy	Theta
p275C^∗^	CP016701	54.922	34.28	62	Dairy	Theta
p275D^∗^	CP016702	54.046	31.77	60	Dairy	Theta
p3107A	CP031539	50.160	35.64	46	Dairy	Theta
p3107B	CP031540	60.216	33.38	56	Dairy	Theta
p3107C	CP031541	26.709	37.63	17	Dairy	Theta
p3107D	CP031542	2.232	33.56	2	Dairy	Theta
p3107E	CP031543	18.170	33.77	13	Dairy	Theta
p3107F	CP031544	4.199	31.60	1	Dairy	Theta
pA12-1	LT599050	5.736	33.68	6	Sourdough	Theta
pA12-2	LT599051	9.105	34.81	9	Sourdough	Theta
pA12-3	LT599052	5.929	34.51	6	Sourdough	Theta
pA12-4	LT599053	69.485	33.35	14	Sourdough	Theta
pAF04	JQ821353	3.801	32.02	4	Dairy	Theta
pAF07	JQ821354.1	7.435	36.44	6	Dairy	Theta
pAF12	JQ821355.1	12.067	33.30	11	Dairy	Theta
pAF14	JQ821356.1	14.419	34.07	11	Dairy	Theta
pAF22	JQ821357.1	22.388	34.95	23	Dairy	Theta
pAG6	AB198069	8.663	33.70	8	Unknown	Theta
pAH33	AF207855	6.159	35.85	7	Dairy	Theta
pAH82	AF243383	20.331	34.44	17	Dairy	Theta
pAR141	DQ288662	1.594	36.14	2	Dairy	RCR
pAW153	HQ646604.1	7.122	31.35	8	Unknown	Theta
pAW601	AJ132009.2	4.752	31.42	1	Unknown	Theta
pBL1	AF242367	10.899	32.62	8	Dairy	Theta
pBM02	AY026767	3.854	35.73	6	Dairy	RCR
pC10A^∗^	CP016703	2.120	34.10	4	Dairy	RCR
pC10B^$^	CP034582	47.093	34.75	48	Dairy	Theta
pC10C^$^	CP034583	7.652	34.88	5	Dairy	Theta
pCD4	AF306799	6.094	33.43	5	Dairy	Theta
pCI305	AF179848	8.694	32.41	8	Dairy	Theta
pCIS1	CP003165	4.263	31.97	2	Dairy	Theta
pCIS2	CP003164	5.461	30.07	4	Dairy	Theta
pCIS3	CP003163	6.159	35.85	5	Dairy	Theta
pCIS4	CP003162	7.045	38.42	10	Dairy	Theta
pCIS5	CP003161	11.676	34.06	10	Dairy	Theta
pCIS6	CP003160	38.673	37.12	30	Dairy	Theta
pCIS7	CP003159	53.051	32.40	48	Dairy	Theta
pCIS8	CP003158	80.592	33.97	72	Dairy	Theta
pCL2.1	U26594	2.047	33.95	2	Unknown	RCR
pCRL1127	AF409136	8.278	34.82	7	Unknown	Theta
pCRL291.1	AF380336	4.640	33.51	3	Unknown	Theta
pCV56A	CP002366	44.098	32.08	41	Human	Theta
pCV56B	CP002367	35.934	34.54	31	Human	Theta
pCV56C	CP002368	31.442	32.49	27	Human	Theta
pCV56D	CP002369	5.543	32.24	6	Human	Theta
pCV56E	CP002370	2.262	33.82	4	Human	Theta
pDBORO	DQ089807	16.404	35.16	15	Unknown	Theta
pDR1-1	AB079381	7.412	33.70	6	Dairy	Theta
pDR1-1B	AB079380	7.344	33.74	6	Dairy	Theta
pFI430	DQ011112.1	59.474	34.63	57	Dairy	Theta
pGdh442	AY849557	68.319	35.11	63	Plant	Theta
pHP003	AF247159	13.433	40.05	6	Dairy	Theta
pIBB477a	CM007354	66.364	33.18	66	Dairy	Theta
pIBB477b	CM007355	64.760	35.10	56	Dairy	Theta
pIBB477c	CM007356	48.496	32.96	42	Dairy	Theta
pIBB477d	CM007357	16.577	31.78	17	Dairy	Theta
pIBB477e	CM007358	11.987	39.6	15	Dairy	Theta
pIL1	HM021326	6.382	32.28	7	Dairy	Theta
pIL105	AF116286	8.506	29.79	7	Dairy	Theta
pIL2	HM021327	8.277	34.82	10	Dairy	Theta
pIL3	HM021328	19.244	35.11	20	Dairy	Theta
pIL4	HM021329	48.978	35.11	47	Dairy	Theta
pIL5	HM021330	23.395	34.49	22	Dairy	Theta
pIL6	HM021331	28.434	33.64	25	Dairy	Theta
pIL7	HM197723	28.546	34.10	26	Dairy	Theta
pJM1A^∗^	CP016747	51.777	35.02	53	Dairy	Theta
pJM1B^∗^	CP016748	48.280	33.94	63	Dairy	Theta
pJM1C^∗^	CP016749	30.146	35.40	29	Dairy	Theta
pJM1D^∗^	CP016750	15.360	35.25	12	Dairy	Theta
pJM1E^∗^	CP016751	11.008	31.95	11	Dairy	Theta
pJM1F^∗^	CP016752	5.329	34.28	6	Dairy	Theta
pJM2A^∗^	CP016742	11.314	37.77	11	Dairy	Theta
pJM2B^∗^	CP016743	13.334	34.48	13	Dairy	Theta
pJM2C^∗^	CP016744	62.261	35.12	56	Dairy	Theta
pJM3A^∗^	CP016737	75.814	35.44	80	Dairy	Theta
pJM3B^∗^	CP016738	47.185	34.84	46	Dairy	Theta
pJM3C^∗^	CP016739	45.257	33.11	59	Dairy	Theta
pJM3D^∗^	CP016740	13.546	33.63	15	Dairy	Theta
pJM3E^∗^	CP016741	3.729	32.90	5	Dairy	Theta
pJM4A^∗^	CP016729	60.219	33.38	74	Dairy	Theta
pJM4B^∗^	CP016730	2.239	33.50	5	Dairy	RCR
pJM4C^∗^	CP016731	5.931	34.53	7	Dairy	Theta
pJM4D^∗^	CP016732	6.207	35.98	8	Dairy	Theta
pJM4E^∗^	CP016733	47.240	34.85	43	Dairy	Theta
pK214	X92946	29.871	32.45	29	Unknown	Theta
pKF147A	CP001835	37.510	32.38	32	Plant	Theta
pKL001	EU289287	6.068	32.86	4	Unknown	Theta
pKP1	FR872378	16.181	35.94	7	Dairy	Theta
pL2	DQ917780	5.299	32.46	5	Dairy	Theta
pLD1	CP020605	8.277	34.82	8	Dairy	Theta
pLD2	CP020606	15.218	34.08	15	Dairy	Theta
pLD3	CP020607	4.242	35.62	2	Dairy	RCR
pLD4	CP020608	12.005	33.51	10	Dairy	Theta
pLD5	CP020609	7.521	33.57	5	Dairy	Theta
pLD6	CP020610	3.363	33.75	2	Dairy	Theta
pLD7	CP020611	30.274	35.17	27	Dairy	Theta
pLP712	FJ649478.1	55.395	37.39	44	Dairy	Theta
pMN5	AF056207	5.670	30.26	4	Dairy	RCR
pMPJM1^∗^	CP016746	193.245	33.83	186	Dairy	Theta
pMPJM2^∗^	CP016745	113.820	34.92	123	Dairy	Theta
pMRC01	AE001272	60.232	30.11	63	Dairy	Theta
pNCDO2118	CP009055	37.571	32.33	32	Plant	Theta
pND324	U44843	3.602	33.37	3	Unknown	Theta
pNP40	DQ534432	64.980	32.33	62	Dairy	Theta
pNZ4000	AF036485	42.810	33.31	45	Dairy	Theta
pQA504	CP003136	3.978	37.83	3	Dairy	Undetermined
pQA518	CP003135	17.661	37.40	13	Dairy	Theta
pQA549	CP003134	49.219	35.14	44	Dairy	Theta
pQA554	CP003133	53.630	34.86	54	Dairy	Theta
pS7a	AJ550509	7.302	33.43	5	Dairy	Theta
pS7b	AJ550510	7.264	33.65	5	Dairy	Theta
pSRQ700	U16027	7.784	34.19	9	Dairy	Theta
pSRQ800	U35629	7.858	31.33	7	Dairy	Theta
pSRQ900	AF001314	10.836	31.13	11	Dairy	Theta
pUC063A^∗^	CP016715	75.962	35.31	79	Dairy	Theta
pUC063B^∗^	CP016716	44.205	34.27	41	Dairy	Theta
pUC063C^∗^	CP016717	11.663	32.55	15	Dairy	Theta
pUC063D^∗^	CP016718	8.697	32.39	10	Dairy	Theta
pUC063E^∗^	CP016719	8.551	31.53	11	Dairy	Theta
pUC06A^∗^	CP016734	36.928	32.10	43	Dairy	Theta
pUC06B^∗^	CP016735	48.632	34.82	55	Dairy	Theta
pUC06C^∗^	CP016736	23.429	31.87	29	Dairy	Theta
pUC06D^$^	CP034579	11.362	31.47	10	Dairy	Theta
pUC06E^$^	CP034580	6.180	33.06	6	Dairy	Theta
pUC06F^$^	CP034581	29.156	34.88	27	Dairy	Theta
pUC08A^∗^	CP016726	89.015	34.19	102	Meat	Theta
pUC08B^∗^	CP016727	49.037	34.22	52	Meat	Theta
pUC08C^∗^	CP016728	15.396	30.83	21	Meat	Theta
pUC08D^∗^	CP034577	5.239	31.00	4	Meat	RCR
pUC08E^∗^	CP034578	7.809	32.81	7	Meat	Theta
pUC109A^∗^	CP016707	64.175	33.17	83	Dairy	Theta
pUC109B^∗^	CP016708	48.261	34.63	51	Dairy	Theta
pUC109C^∗^	CP016709	11.868	32.20	14	Dairy	Theta
pUC109D^∗^	CP016710	11.333	31.64	13	Dairy	Theta
pUC109E^∗^	CP016711	4.237	33.35	5	Dairy	Theta
pUC109F^∗^	CP016712	2.413	33.11	3	Dairy	RCR
pUC109G^$^	CP034576	25.328	34.40	21	Dairy	Theta
pUC11A^∗^	CP016720	59.284	33.91	65	Meat	Theta
pUC11B^∗^	CP016721	49.307	34.22	53	Meat	Theta
pUC11C^∗^	CP016722	19.351	35.19	18	Meat	Theta
pUC11D^∗^	CP016723	15.393	30.82	17	Meat	Theta
pUC11E^$^	CP034572	7.809	33.12	8	Meat	Theta
pUC11F^∗^	CP016725	5.238	30.99	4	Meat	RCR
pUC77A^∗^	CP016713	6.083	35.75	7	Dairy	Theta
pUC77B^∗^	CP016714	63.462	34.86	66	Dairy	Theta
pUC77C^$^	CP034573	62.882	36.14	58	Dairy	Theta
pUC77D^$^	CP034574	39.604	33.93	47	Dairy	Theta
pUC77E^$^	CP034575	6.153	35.79	7	Dairy	Theta
pUL8A^∗^	CP016704	7.652	33.95	6	Dairy	Theta
pUL8B^∗^	CP016705	27.296	35.31	30	Dairy	Theta
pUL8C^∗^	CP016706	2.119	34.07	3	Dairy	RCR
pVF18	JN172910	18.977	33.90	21	Dairy	Theta
pVF21	JN172911	21.728	33.59	14	Dairy	Theta
pVF22	JN172912	22.166	35.14	19	Dairy	Theta
pVF50	JN225497	53.876	34.50	41	Dairy	Theta
pWC1	L75827	2.846	29.48	1	Dairy	RCR
pWV01	X56954	2.178	33.43	4	Dairy	RCR
pWVO2	NC_002193.1	3.826	31.34	1	Unknown	Theta
SK11 p1	CP000426	14.041	34.37	13	Dairy	Theta
SK11 p2	CP000427	9.554	30.44	10	Dairy	Theta
SK11 p3	CP000428	74.750	35.41	69	Dairy	Theta
SK11 p4	CP000429	47.208	34.84	42	Dairy	Theta
SK11 p5	CP000430	14.206	33.55	10	Dairy	Theta

*^∗^Plasmids sequenced in the context of the current study (PacBio SMRT). ^$^Plasmids sequenced in the context of the current study (Illumina MiSeq).*

In parallel with SMRT sequencing, an Illumina-based approach was applied to the sixteen lactococcal strains to identify strains where plasmids were potentially absent from the completed assemblies. Re-sequencing of genomes was performed on an Illumina MiSeq platform (executed by GenProbio S.R.L., Parma, Italy), to an average coverage of ∼100–125×. Sequences obtained were first quality checked using IlluQC.pl from the NGS QC Toolkit (v2.3) (Patel and Jain, 2012) and assembled with AbySS (v1.9.0) (Simpson et al., 2009). Based on whole genome alignments contigs absent from the SMRT assemblies were identified. Remaining low quality regions and sequence conflicts were then resolved by primer walking and Sanger sequencing of PCR products (performed by Eurofins MWG Operon, Germany).

### General Feature Predictions

Annotation of plasmid sequences was performed on both newly sequenced and publically available plasmid sequences using the following protocol. ORF prediction, defined as a continuous stretch of codons without a stop codon was performed with Prodigal v2.5 prediction software^1^ with a general minimum cut-off of >50 bp and confirmed using BLASTX v2.2.26 alignments (Altschul et al., 1990). ORFs were automatically annotated using BLASTP v2.2.26 (Altschul et al., 1990) analysis against the non-redundant protein databases curated by the National Centre for Biotechnology Information (NCBI)^2^. Artemis v16 genome browser and annotation tool was used to manually curate identified ORFs^3^ and for the combination and inspection of ORF results. The final ORF annotations were refined where necessary using additional software tools and database searches, such as Pfam (Bateman et al., 2004), Uniprot/EMBL^4^ and Bagel3 (Van Heel et al., 2013).

### Pan-Plasmidome Analysis

Pan-plasmidome analysis was performed utilizing the PGAP v1.0 pipeline (Zhao et al., 2012) according to Heaps law pan-genome model (Tettelin et al., 2005). The ORF content of each plasmid was organized into functional gene clusters via the Gene Family method. ORFs which produced an alignment with a minimum of 50% sequence identity across 50% of the gene or protein length (both nucleotide and amino acid sequences are applied in parallel) were clustered and a pan-plasmidome profile was subsequently generated (Tettelin et al., 2005).

### Comparative Genomics

Tandem Repeats Finder v4.02 (Benson, 1999) was applied to identify nucleotide tandem repeats at a potential plasmid origin of replication. Plasmids were assigned to be employing a Theta mode of replication where the gene encoding replication protein is preceded by 3.5 iterations of a 22 bp tandem repeat with an A/T rich 10 bp direct repeat located further upstream (Kiewiet et al., 1993). Alternatively, plasmids that replicate by rolling circle replication (RCR) can be identified because they rely on a replication protein and a double-stranded origin of replication (dso). Putative dso replication sites were identified based on nucleotide conservation to previously identified dso’s, containing a *nic* site composed of one or more inverted repeats, and a Rep-binding site consisting of 2–3 direct repeats or an inverted repeat (Del Solar et al., 1993; Mills et al., 2006).

All sequence comparisons at protein level were performed via all-against-all, bi-directional BLAST alignments (Altschul et al., 1990). An alignment cut-off value of >50% amino acid identity across 50% of the sequence length was used (with an associated E-value of <0.0001). For analysis and clustering of these results, the MCL was implemented in the mclblastline pipeline v12-0678 (Enright et al., 2002). TM4 MeV, MultiExperiment Viewer v4.9 was used to view MCL clustering data and conduct hierarchal clustering (HCL)^5^. The HCL analysis was exported from TM4 MeV in Newick tree format and visualized using ITOL (Interactive Tree of Life) (Letunic and Bork, 2016).

### Pulsed Field Gel Electrophoresis (PFGE)

*Lactococcus lactis* subsp. *cremoris* strains JM1 and JM2 were cultured in M17 broth (Oxoid) supplemented with 0.5% (w/v) lactose at 30°C without agitation overnight. PFGE plugs were then prepared and restricted with SI nuclease (Thermo Fisher Scientific, Ireland) as previously described (Bottacini et al., 2015).

A 1% (wt/vol) PFGE agarose gel was prepared in 0.5X TBE [89 mM Tris-borate, 2 mM EDTA (pH 8.3)] buffer and the PFGE plugs were melted in and sealed with molten agarose in 0.5X TBE buffer. A CHEF-DR III pulsed-field system (Bio-Rad Laboratories, Hercules, CA, United States) was used to resolve the DNA fragments at 6 V/cm for 18 h in 0.5X TBE running buffer maintained at 14°C with linear increment (interpolation) of pulse time from 3 to 50 s. DNA ladder (Chef DNA lambda) was included in each gel (number 170-3635; Bio-Rad Laboratories). The gels were stained in ethidium bromide (10 mg/ml) (25 μl/500 ml dH_2_O) for 120 min under light-limited conditions and destained in distilled water for 60 min. Gels were visualized by UV transillumination.

### Bacteriocin Assays

Lactococcal strains were cultured in M17 broth (Oxoid) supplemented with 0.5% (w/v) lactose or glucose (strain-dependent) at 30°C without agitation overnight. 3 μl of overnight culture was spotted on M17 agar supplemented with 0.5% (w/v) glucose and left at 30°C overnight. Cells that had grown on the spotted areas were inactivated by exposure to UV light for 30 min. Plates were then overlaid with a semi-solid M17 agar (0.4% agarose) containing indicator strain *L*. *lactis* HP. Zones of inhibition were visualized and measured after 24 h.

### Genbank Accession Numbers of Applied Strains

*Lactococcus lactis* subsp. *lactis* IL1403: AE005176; *L*. *lactis* subsp. *lactis* IO-1: AP012281; *L*. *lactis* subsp. *lactis* 184: CP015895; *L*. *lactis* subsp. *lactis* 229: CP015896; *L*. *lactis* subsp. *lactis* 275: CP015897; *L*. *lactis* subsp. *lactis* UC06: CP015902; *L*. *lactis* subsp. *lactis* UC08: CP015903; *L*. *lactis* subsp. *lactis* UC11: CP015904; *L*. *lactis* subsp. *lactis* UC063: CP015905; *L*. *lactis* subsp. *lactis* UC77: CP015906; *L*. *lactis* subsp. *lactis* UL8: CP015908; *L*. *lactis* subsp. *lactis* C10: CP015898; *L*. *lactis* subsp. *cremoris* SK11: CP000425; *L. lactis* subsp. *cremoris* MG1363: AM406671; *L*. *lactis* subsp. *cremoris* NZ9000: CP002094; *L*. *lactis* subsp. *cremoris* A76: CP003132; *L*. *lactis* subsp. *cremoris* UC509.9: CP003157; *L*. *lactis* subsp. *cremoris* KW2: CP004884; *L*. *lactis* subsp. *cremoris* 158: CP015894; *L*. *lactis* subsp. *cremoris* UC109: CP015907; *L*. *lactis* subsp. *cremoris* JM1: CP015899; *L*. *lactis* subsp. *cremoris* JM2: CP015900; *L*. *lactis* subsp. *cremoris* JM3: CP015901; *L*. *lactis* subsp. *cremoris* JM4: CP015909; *L*. *lactis* subsp. *cremoris* 3107: CP031538; *L*. *lactis* subsp. *cremoris* IBB477: CM007353; *L*. *lactis* subsp. *lactis* A12: LT599049; *L*. *lactis* subsp. *lactis* biovar. *diacetylactis* FM03: CP020604; *L*. *lactis* subsp. *lactis* 14B4: CP028160; and *L*. *lactis* subsp. *cremoris* HP: JAUH00000000.1.

## Results

### Plasmid Sequencing

In this study the sequences of 83 plasmids were elucidated utilizing a combined PacBio SMRT sequencing and Illumina MiSeq approach, and represent the detected plasmid complement of 16 lactococcal genomes (Kelleher et al., 2017). Initially 69 plasmids were identified from the SMRT sequencing data by modifying the RS_HGAP_assembly protocol in SMRT portal to a reduced minimum coverage cut-off of 15-fold coverage. To ensure complete coverage of the full plasmid complement the complete genomes of all 16 strains were re-sequenced utilizing an Illumina MiSeq approach which resulted in the eludication of a further 14 plasmids (indicated in Table 1) that had not been detected based on the original SMRT assemblies. These 14 plasmids ranged in size from 6 to 62 Kbp, indicating that their absence from the SMRT dataset was in the majority of cases not associated with exclusion from the library based on their small size. Therefore, it was hypothesized that the absence of some plasmids from the SMRT dataset was either due to a lower plasmid copy number (SMRT library preperation does not incorporate an amplification step) or due to a bias in the DNA extraction protocol. Conversely, no plasmids present in the SMRT assemblies, were absent from the Illumina data, however, Illumina sequencing generated heavily fragmented assemblies (∼100–250 contigs per strain), making eludication of complete plasmid sequences, particular for larger plasmids significantly more challenging if not impossible. The main advantage of SMRT technology is the long read length it achieves. Due to the high frequency of repetitive transposable elements, assembly of lactococcal genomes and plasmids is cumbersome. SMRT sequencing was shown to be very useful in obtaining reliable and accurate assemblies, being particularly beneficial for assembling larger lactococcal plasmids which frequently possess a mosaic type structure and contain multiple identical IS elements (Ainsworth et al., 2014c). Therefore, a combined sequencing approach is suggested as the most effective strategy for the complete sequencing of lactococcal strains.

### General Plasmid Features

The sequenced plasmid dataset was combined with a further one hundred and seven plasmids retrieved from the NCBI database (National Centre for Biotechnology Information) (Table 1). In total, the features of one hundred and ninety plasmids derived from fifty three lactococcal strains in addition to seventeen lactococcal plasmids without an assigned strain were investigated. This extra-chromosomal DNA complement amounts to 4,987 Kbp of DNA and is predicted to represent 4,905 CDSs (i.e., ORFs that encode protein products), thus contributing very substantially to the overall genetic content of *L*. *lactis*.

The vast majority of currently sequenced plasmids originate from strains that were isolated from the dairy niche (149 out of 190 analyzed plasmids). These dairy lactococci carry between one and twelve plasmids (the latter in *L. lactis* biovar. diacetylactis FM03P), accounting for up to 355 Kbp of extra-chromosomal DNA in a given strain (as is the case for *L. lactis* JM1). The size of individual lactococcal plasmids varies widely from the smallest *L*. *lactis* KLDS4.0325 plasmid 2, with a size of 0.87 Kbp, to the two megaplasmids, each maintained by *L*. *lactis* JM1 and *L*. *lactis* JM2, with a size of 193 and 113 Kbp, respectively. The GC content of lactococcal plasmids ranges from ∼30–38%, whilst the average GC content of previously sequenced chromosomes is more constrained (34–36%). Only three lactococcal plasmids deviate from this range; pWC1 29.48, pIL105 29.79, and pHP003 40.05%, where the latter is closer to *Streptococcus thermophilus* genomic GC-content, which ranges from 39 to 40% (Fernández et al., 2011).

Lactococcal plasmids are known to replicate via either of two alternative methods, RCR or theta-type replication (Mills et al., 2006; Ainsworth et al., 2014c). Based on predicted plasmid replication proteins/origins it appears that the majority of lactococcal plasmids (174 of the current data-set) replicate via the theta-type mechanism, while only a small proportion appears to utilize RCR (sixteen of the current data-set). The relatively small number of plasmids utilizing RCR may be attributed to a number of factors, such as the fact that RCR plasmids can only support a limited replicon size (<10 Kbp), incompatibility with other RCR type plasmids (Leenhouts et al., 1991), and/or intrinsic structural and segregational instability (Ainsworth et al., 2014c). In three instances, the analysis identified plasmids for which the replication mode could not be clearly determined as the origin of replication of these plasmids did not conform to the typical origin of replication associated with RCR or theta replication.

### Pan-Plasmidome Calculation

**FIGURE 1:**
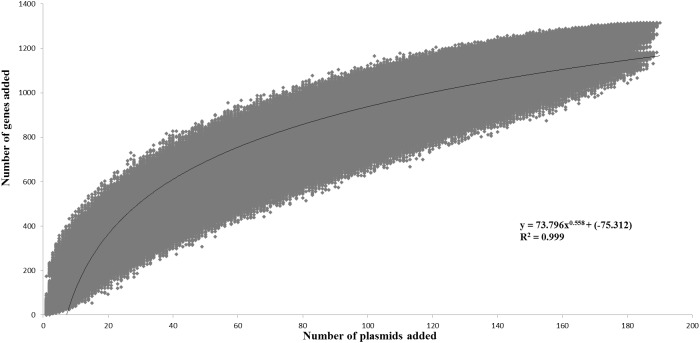
Pan-plasmidome of *Lactococcus lactis*. This graph represents the accumulated number of new genes in the *L. lactis* pan-plasmidome plotted against the number of plasmids added. The deduced mathematical equation is also indicated.

The pan-plasmidome calculation provides an overview of the overall genetic diversity of the *L*. *lactis* plasmidome, the latter representing the total plasmid content harbored by (sequenced) members of the *L*. *lactis* taxon. To calculate the pan-plasmidome, a pan-genome analysis approach was applied using the PGAP v1.0 pipeline (Zhao et al., 2012). The resultant pan-plasmidome graph (Figure 1) displays an asymptotic curve rising steadily as each of the one hundred and ninety plasmids included in the analysis is added until a total pan-plasmidome size of 1, 315 CDSs was reached. The trend observed in the pan-genome indicates that the pan-plasmidome remains in a fluid or open state, and that, therefore, continued plasmid sequencing efforts will further expand the observed genetic diversity among lactococcal plasmids. The PGAP pipeline was also used to determine the core genome of the lactococcal plasmid sequence data set. Interestingly, no single CDS is conserved across all plasmids resulting in an empty core genome.

The *L*. *lactis* pan-genome, based on chromosomal sequences only, has previously been calculated to constitute 5,906 CDSs (Kelleher et al., 2017). When compared with the calculated lactococcal plasmidome (1,315 CDSs), it is obvious that the lactococcal plasmidome contributes very substantially to overall lactococcal genetic diversity.

### MCL Analysis of the Pan-Plasmidome

To explore the genetic content of the one hundred and ninety plasmids employed in this study, all-against-all reciprocal BLASTP analysis and MCL (Markov clustering) was conducted (Altschul et al., 1990; Enright et al., 2002). The plasmidome was determined to comprise 885 protein families, of which 413 represented single member protein families, evidence of the divergent nature of the plasmid sequences. Furthermore, 421 of these families constitute hypothetical protein families, being represented by a total of 1,341 individual proteins. These hypothetical proteins encompass 22.7% of the total CDSs in the lactococcal plasmidome.

The second largest constituent of the lactococcal plasmidome is that represented by transposable elements. Transposable elements encompass 825 CDS, or 15.7% of the plasmidome, with members of the IS6, IS30, IS982, and ISL3 insertion families being among the most dominant genetic elements. These mobile elements are responsible for the transfer and recombination of DNA (Nicolas et al., 2007; Machielsen et al., 2011; Alkema et al., 2016) and are likely to contribute to a fluid lactococcal plasmidome.

**FIGURE 2:**
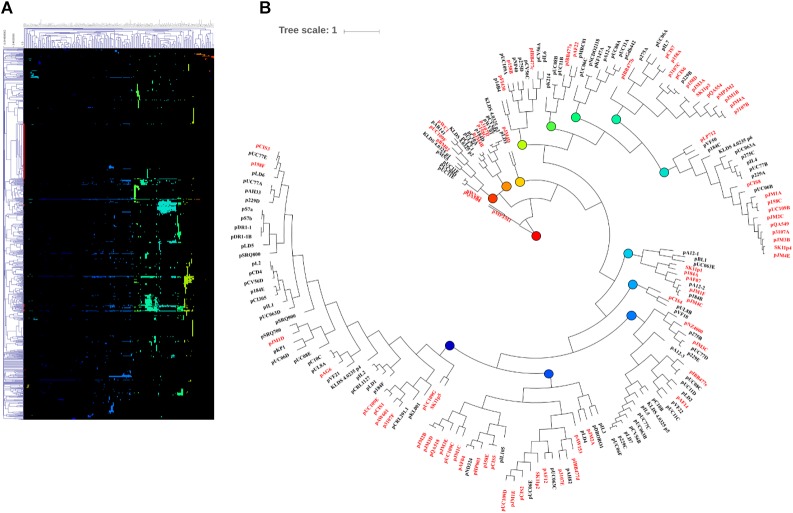
HCL analysis of the lactococcal plasmidome. Hierarchical clustering analysis **(A)** representing the presence/absence of gene families from 190 lactococcal plasmids. Absence of gene family members is indicated in black and presence of gene family members is indicated in color. **(B)** Circular tree representation of panel **A** displaying HCL plasmid groupings. Plasmids from subsp. *cremoris* strains are labeled in red while plasmids from subsp. *Lactis* strains are labeled in black. Colored nodes correspond to the presence of genes (colors) in HCL matrix (panel **A**).

Following MCL analysis, HCL of the pan-plasmidome was used to cluster plasmids based on their genetic content (Figure 2). The high level of diversity within the pan-plasmidome is demonstrated by the observed disparity within the HCL matrix. HCL analysis resulted in thirteen clusters with three outliers; pMPJM1, pWVO2, and pQA504 (Figure 2B). Plasmid pWVO2 encodes a single replication gene, pQA504 contains three CDS (*rep* gene, *mob* gene, and hypothetical gene), while pMPJM1 encodes 188 CDS and shares little homology with other lactococcal plasmids. The remaining thirteen clusters did not display subspecies specificity, each cluster containing plasmids from both subsp. *lactis* and subsp. *cremoris* hosts.

### Lactococcal Megaplasmids

**FIGURE 3:**
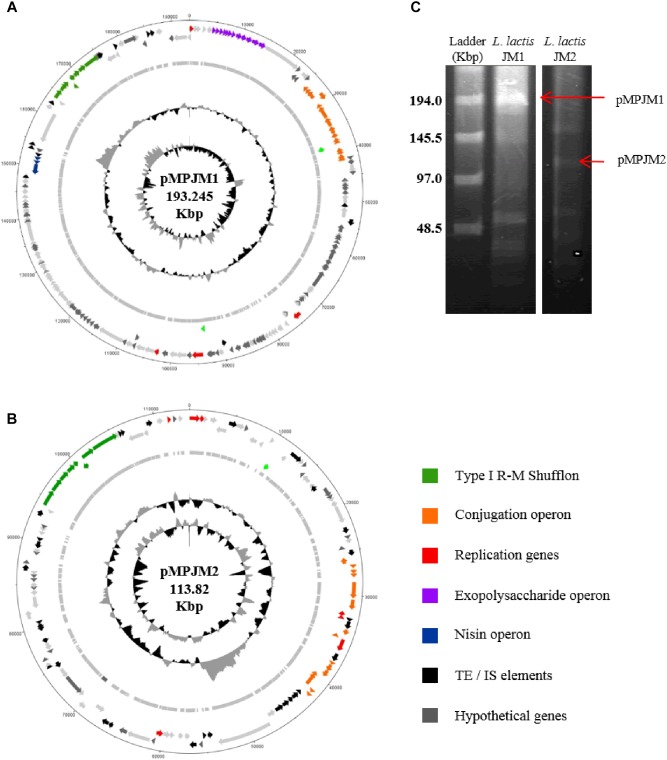
General features of megaplasmids pMPJM1 and pMPJM2. Circular maps of **(A)** pMPJM1 and **(B)** pMPJM2. CDS of interest are highlighted in color. **(C)** PFGE image of pMPJM1 (lane 2) and pMPJM2 (lane 3), the possible position of each of the two megaplasmids is indicated by a red arrow. CHEF lambda (Bio-Rad Laboratories, Hercules, CA, United States) DNA ladder is displayed in lane 1.

Typically, *L*. *lactis* plasmids range in size from 1 to 50 Kbp, and, prior to this study, the largest plasmid identified in *L*. *lactis* was the self-conjugative mega-plasmid of 155,960 bp in *L*. *lactis* subsp. *lactis* bv. *diacetylactis* S50 (Kojic et al., 2005). *L*. *lactis* S50 p7 represents the first lactococcal megaplasmid and encodes genes for Proteinase PI and lactococcin A and is part of a larger plasmid complement of 7 plasmids totaling 336 Kbp (Kojic et al., 2005). Recently (May 2018) the plasmid complement of *L*. *lactis* subsp. *lactis* KLDS 4.0325 (Yang et al., 2013) has been updated in the public NCBI data base with three additional plasmid sequences, the largest plasmid measuring 109 Kbp (plasmid 6). In the current study, whole genome sequencing efforts resulted in the identification of two plasmids that were larger than 100 Kbp, namely pMPJM1 (193 Kbp) and pMPJM2 (113 Kbp) from *L*. *lactis* JM1 and *L*. *lactis* JM2, respectively, and owing to their size are defined as megaplasmids (Anton et al., 1995; Barton et al., 1995; Figure 3A,B). Pulsed field gel electrophoresis also identified bands which would be consistent with plasmids of that size, although unambiguous validation will require Southern hybridization analysis (Figure 3C).

The larger of the two megaplasmids, pMPJM1, encompasses 186 CDSs and is presumed to replicate (as expected for such a large replicon) via the theta-type replication mechanism [based on the identification of the origin of replication (*ori*), comprised of an AT-rich region plus three and a half iterons of 22 bp in length] (Seegers et al., 1994). pMPJM1 encompasses, among others, gene clusters predicted to be responsible for (exo)polysaccharide biosynthesis, conjugation and nisin resistance, while it also specifies an apparently novel type I RM shufflon system (as well as a high proportion of unique/hypothetical CDSs). The overall sequence of the plasmid shows little homology to previously sequenced plasmids in the NCBI databases, however, it shares 24% sequence coverage with 99% nucleotide identity to the other identified megaplasmid pMPJM2, which indicates that they share a common ancestor. pMPJM2 encodes 123 CDSs and BLASTN analysis identified sequence identity to a number of different lactococcal plasmids indicating a mosaic genetic structure commonly seen in large lactococcal plasmids (Ainsworth et al., 2014c). pMPJM2 also encodes a putative conjugation operon and a very close homolog of the type I RM shufflon system of pMPJM1. The third lactococcal megaplasmid KLDS 4.0325 plasmid 6 (109 Kbp) encodes 119 CDSs including the *lac* operon and associated *opp* oligopeptide uptake system.

### Technological Properties

Strains of *L. lactis* are commonly used as starter cultures employed by the dairy industry (Beresford et al., 2001), and their dairy adaptations such as citrate metabolism and lactose utilization are frequently plasmid-encoded. In *L*. *lactis*, citrate uptake and subsequent diacetyl production is governed by the plasmid-encoded *citQRP* operon (Drider et al., 2004; Van Mastrigt et al., 2018b). In the current data set, only four plasmids contain the *citQRP* operon, *L. lactis* CRL1127 plasmid pCRL1127, *L. lactis* IL594 plasmid pIL2 (Górecki et al., 2011), *L. lactis* FM03 plasmid pLD1 and *L. lactis* 184 plasmid p184F. However, the latter operon in p184F appears to lack *citQ* which encodes a leader peptide. Lactose metabolism is controlled by the *lac* operon consisting of the genes *lacABCDEFGX* and is regulated by a repressor, encoded by the adjacent *lacR* gene (Cords et al., 1974), both citrate and lactose utilization have previously been described in detail (Cords et al., 1974; Górecki et al., 2011).

The *lac* operon was found to be present on twenty four plasmids (in 24 different strains) (Table 2). The plasmids analyzed were derived from 53 lactococcal strains in addition to 17 lactococcal plasmids unassigned to a particular strain, and represented the total plasmid complement of 26 such strains. In all cases bar two, the strains were isolated from the dairy environment with the exception of *L*. *lactis* NCDO1867 isolated from peas and *L. lactis* KLDS 4.0325 isolated from fermented food (Table 1). Alternative lactose metabolism methods have previously been observed in *L*. *lactis*. For example, *L. lactis* MG1363 does not harbor the *lac* operon, yet is capable of growth on lactose-supplemented media due to the activity of a cellobiose-specific phosphotransferase system (PTS), which can act as an alternative lactose utilization pathway (Solopova et al., 2012). Another example of an alternative lactose metabolic pathway is found in the slow lactose fermenter *L. lactis* NCDO2054, which metabolizes lactose via the Leloir pathway (Bissett and Anderson, 1974). Plasmid integration events have also resulted in the integration of the *lac* operon in the chromosome of *L*. *lactis* SO, where it is located 20 Kbp downstream of an integrated *opp* operon, sharing significant homology with (the *lac* operons of) plasmids pCV56B, pSK08, pKF147A, and pNCDO2118 (Kelleher et al., 2017). Due to the lack of sequencing projects that report fully sequenced genomes, defining the true frequency of lactose utilization is challenging. However, of those strains for which complete genome sequencing projects have been described [30 strains in Kelleher et al. (2017)], 22 were found to be capable of metabolizing lactose based on growth in lactose supplemented broth, 19 via plasmid-encoded *lac* operons, one via a chromosomally encoded *lac* operon and two by an alternative pathway. This analysis included 12 subsp. *cremoris* strains, of which all but one possessed genes for a lactose utilization mechanism, the exception being strain KW2, which lacks a plasmid complement.

**Table 2:** Overview of presence of plasmid-encoded *lac*/*opp* operons.

Stain	Subspecies	Origin	Plasmid
SK11	*cremoris*	Dairy	pSK114
158	*cremoris*	Dairy	p158C
229	*lactis*	Dairy	p229A
275	*lactis*	Dairy	p275C
3107	*cremoris*	Dairy	p3107A
A76	*cremoris*	Dairy	pQA549
CV56	*lactis*	Dairy	pCV56A
IBB477	*cremoris*	Dairy	pIBB477c
JM1	*cremoris*	Dairy	pJM1A
JM2	*cremoris*	Dairy	pJM2C
JM3	*cremoris*	Dairy	pJM3B
JM4	*cremoris*	Dairy	pJM4E
KLDS 4.0325	*lactis*	Fermented food	p6
UC063	*lactis*	Dairy	pUC063A
UC06	*lactis*	Dairy	pUC06B
UC109	*cremoris*	Dairy	pUC109B
UC77	*lactis*	Dairy	pUC77B
UC509.9	*cremoris*	Dairy	pCIS8
DPC3901	*lactis* bv. diacetylactis	Dairy	pVF50
IL594	*lactis*	Dairy	pIL4
NCDO712	*cremoris*	Dairy	pLP712
UC08	*lactis*	Dairy	pUC08A
UC11	*lactis*	Dairy	pUC11A
NCDO1867	*lactis*	Plant	pGdh442

### Conjugation

Conjugation and transduction are believed to be the dominant mechanisms of plasmid transfer in *L*. *lactis* (Ainsworth et al., 2014c). Particular emphasis has been placed on conjugation as it is considered a naturally occurring DNA transfer process and for this reason may be used in food-grade applications to confer beneficial traits to industrial strains (Mills et al., 2006). Generally, during conjugation the AT-rich, so-called “origin of transfer” or *oriT* of the conjugative plasmid is nicked by a nickase, and the resulting ssDNA strand is passed to a recipient cell (Grohmann et al., 2003). The *tra* (transfer) locus is believed to be responsible for the donor-to-recipient DNA transfer process of conjugation, though the precise mechanistic details of the conjugation process in *L*. *lactis* has not yet been fully elucidated. Plasmids which do not encode the *tra* operon, may also be co-transferred by conjugation in instances where a plasmid contains an *oriT* sequence and at least one mobilization gene (*mobA*, *B*, *C*, or *D*). Additional genes can also be involved in conjugation in *L*. *lactis*; an example of this is *cluA*, which encodes a cell surface-presented protein, and which is involved in cell aggregation and thought to be essential for high efficiency conjugal transfer (Stentz et al., 2006). Furthermore, a chromosomally associated, so-called sex factor in *L*. *lactis* has been shown to facilitate transfer of chromosomal genes during conjugation (Gasson et al., 1995).

**FIGURE 4:**
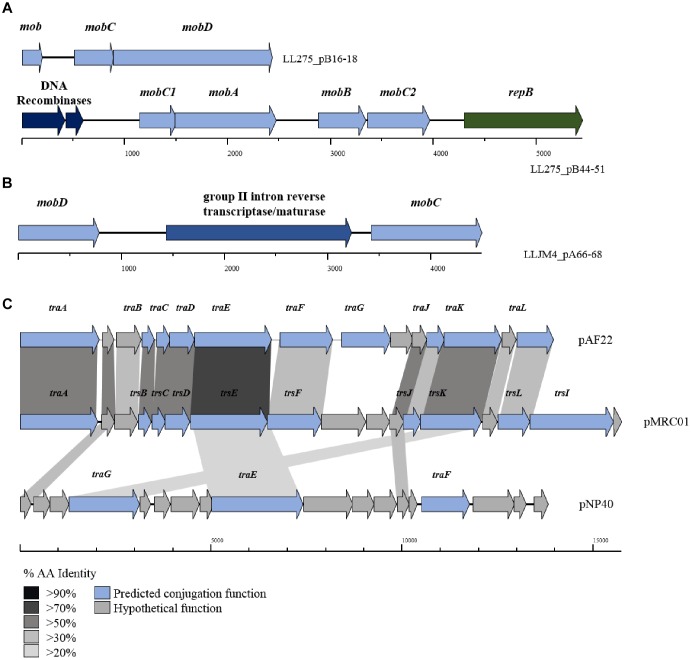
BLAST map of active lactococcal conjugation gene clusters. The image describes the genetic organization of mob genes in **(A)** p275B, **(B)** pJM4A, and **(C)** the conjugation gene clusters from plasmids pAF22, pMRC01, and pNP40. All three plasmids have previously been shown to be self-transmissible by conjugation. Gene synteny is highly conserved between pAF22 and pMRC01, but amino acid identity is not, while pNP40 represents a divergent system. Amino acid identity is indicated by the shaded boxes. Arrows colored blue indicate predicted conjugative function, while arrows shaded gray indicate hypothetical functions.

The *tra* locus, which encodes the protein complex responsible for donor-to-recipient DNA transfer has as yet been fully eludicated. Previous studies have identified the role of *traF* as encoding a membrane-spanning protein involved in channel formation and membrane fusion. In addition, the *traE* and *traG* genes have been proposed to encode proteins involved in the formation of the conjugal pilus similar to type IV secretion systems (O’Driscoll et al., 2006; Górecki et al., 2011). Typically, the three *tra* genes (i.e., *traE*, *traF*, and *traG*) are part of a larger gene cluster (consisting of up to 15 genes; Figure 4), including *traA*, which encodes a DNA relaxase. In the current data set, 34 genes with homology to *traG* were identified on 27 plasmids (present in duplicate on seven plasmids) along with five occurrences of *traE/F* also being present (in the case of plasmids pIBB477A, pUC08B, pUC11B, pAF22, and pMRC01).

The precise functions for the remainder of the genes in the *tra* gene cluster have yet to be elucidated, though additional *tra*-encoded functions have been predicted in a small number of cases, the majority based on homology to the *trs* operon in *Staphylococcus* (Sharma et al., 1994). For example, *traJ* and *traL* were identified on plasmids pAF22, pIBB477a and pMRC01, and *traB*, *traC*, *traD*, *traF* (mating channel formation) and *traK* (P-loop NTPase) on plasmids pUC08B, pIBB477a, pUC11B, pAF22, and pMRC01. Plasmids pAF22, pMRC01, and pNP40 have all previously been demonstrated to be capable of conjugation (Harrington and Hill, 1991; Coakley et al., 1997; O’Driscoll et al., 2006; Fallico et al., 2012). However, the annotation(s) of the operons involved in conjugation is not well defined and they are currently poorly characterized. This is also amplified by both a lack of sequence conservation and limited synteny within the genes that make up these conjugation-associated genetic clusters (Figure 4).

While the *tra* operon is thought to be responsible for the formation of conjugal pili, previous studies have identified a number of genes believed to play a role in the mobilization of other (non-self-transmissible) plasmids in *L*. *lactis* (Mills et al., 2006; O’Driscoll et al., 2006; Millen et al., 2012); principal among these are the *mob* (mobilization) genes. Mobilization genes are responsible for nicking the plasmid’s dsDNA at a particular site and forming a relaxosome, which allows the transfer of a single stranded template to a recipient cell. Variants of four main *mob* genes are distributed throughout the lactococcal plasmidome; *mobA* and *mobD* encode nickases, and *mobB* and *mobC*, whose protein products are thought to form a relaxosome with an associated nickase (either *mobA* or *mobD*) are typically present in the genetic configuration *mobABC* or *mobDC*. Comparative analysis identified 422 occurrences of *mob* genes (any of the afore mentioned *mob* genes) distributed across the 190 plasmids assessed in this study, including 15 occurrences of a predicted retron-type reverse transcriptase or maturase (located between *mobD* and *mobC*) believed to play a role in DNA recombination. The results indicate that 59.5% of plasmids in the lactococcal plasmidome carry at least one or more genes encoding mobilization proteins.

The lactococcal megaplasmids pMPJM1 and pMPJM2 harbor two (16 Kbp) regions putatively involved in conjugation and/or mobilization. In the case of pMPJM2 the predicted region was found to contain homologs of *mobC* and *mobD*, encoding a nickase and an associated relaxase near a possible secondary replication origin. However, the presence of five transposase-encoding genes and the lack of predicted *tra* genes with conserved functions suggest that this plasmid is not capable of autonomous conjugation (though mobilization is possible).

**FIGURE 5:**
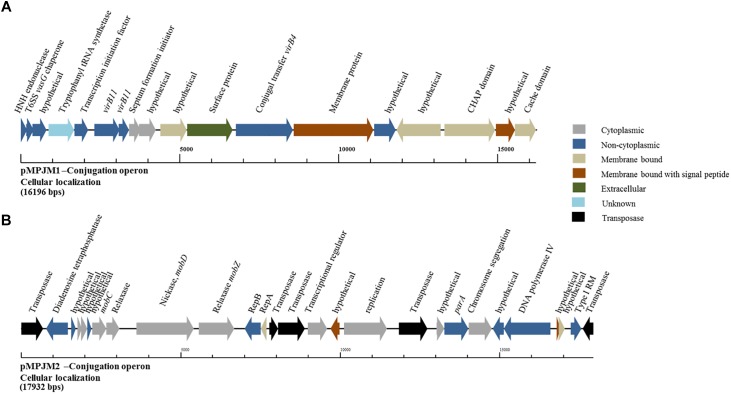
Genetic organization of the putative conjugation gene clusters in pMPJM1 and pMPJM2. **(A)** Represents the putative conjugation locus in pMPJM1. **(B)** Represents the putative conjugation locus in pMPJM2. Colors indicate the predicted cellular localization of each product. The system in pMPJM1 appears to encode proteins involved in conjugal transfer, while the cellular localization data is predictive of a transmembrane complex. Conversely, the conjugation locus in pMPJM2 appears to be involved in mobilization rather than conjugation, and the presence of a number of insertion elements suggest it is unlikely to be functional.

Conversely, analysis of pMPJM1 identified a more divergent system to that typically found in lactococcal plasmids. Three hypothetical proteins were found to contain the PFAM domain usually conserved in conjugation proteins (pfam12846), in addition to a homolog of *virB11*, whose deduced product acts as a type IV secretory pathway ATPase (pfam00437). Cellular localization analysis of the operon using PsortB was also indicative of a transmembrane complex composed of cytoplasmic, membrane bound, signal and extracellular proteins (Figure 5). The divergence of both operons from typical lactococcal conjugative operons suggests that these two megaplasmids have lost their conjugative ability or may possess a conjugation system with very few identifiable similarities to currently known systems.

### Cell Surface Interactions (Adhesion & EPS)

Mucin-binding proteins, i.e., those allowing adhesion to the mucin layer of the gastrointestinal tract, are considered essential for stable and extended gut colonization by LAB (Von Ossowski et al., 2010). While lactococci are typically not associated with the human gut and do not have a growth temperature profile that would be inconsistent with GIT colonization., instances of such proteins encoded by lactococcal plasmids have been reported (Kojic et al., 2011; Lukić et al., 2012; Le et al., 2013). Muco-adhesive proteins are considered of paramount importance for the efficacy of probiotic bacteria (Von Ossowski et al., 2010) and the presence of such elements in *L*. *lactis* may have significant commercial impact for their role in functional foods. Analysis of the plasmids assessed in our study identified a number of strains with predicted novel muco-adhesive elements, similar to those found in pKP1 (Kojic et al., 2011). Plasmid pKP1 encodes two proteins, a mucin-binding domain-containing protein and an aggregation-promoting protein AggL, which promotes its binding to colonic mucosa (Lukić et al., 2012). While no direct homolog of AggL was detected, mucus-binding protein-encoding genes were identified on plasmids p14B4, p275A, p275B, pUC08B, and pUC11B perhaps reflecting a potential for gastrointestinal persistence conferred to the strains that carry these plasmids. A number of additional proteins predicted to be host cell surface-associated were detected during the analysis. For example, pUC11C encodes two class C sortases, which are commonly involved in pilus biosynthesis (Von Ossowski et al., 2010; Lebeer et al., 2012), while p275A encodes an LPXTG anchor domain, cell surface-associated protein. Interestingly, each of these strains belongs to subspecies *lactis* and is capable of growth at 37°C, which would impede growth of their *cremoris* counterparts, which are generally less thermo-tolerant. *L*. *lactis* JM1 is the sole *cremoris* strain that is predicted to encode proteins directly involved in host cell surface alterations. This plasmid encodes five putative proteins containing a 26-residue repeat domain found in predicted surface proteins (often lipoproteins) and one collagen-binding domain protein.

The plasmid encoded lactococcal cell wall anchored proteinase, PrtP, involved in the breakdown of milk caseins in dairy lactococci, has previously been shown to cause a significant increase in cell adhesion to solid glass and tetrafluoroethylene surfaces (Habimana et al., 2007). More recently, *L*. *lactis* subsp. *cremoris* IBB477 was found to contain two plasmids, pIBB477a and pIBB477b, which encode cell wall-associated peptidases that have been shown to mediate adhesion to bare mucin and fibronectin coated polystyrene and HT29-MTX cells (Radziwill-Bienkowska et al., 2017). Analysis of the current data-set which contains a large number of dairy derived plasmids, identified a further 194 CDS homologous to the cell wall-associated peptidase S8 (PrtP) of IBB477. Whilst extracellular cell wall proteinases have been shown to be directly associated with the bitter flavor defect in Cheddar cheese varieties (Broadbent et al., 2002), a potential role for these peptidases in gut adhesion may present a more positive view of these elements.

**FIGURE 6:**
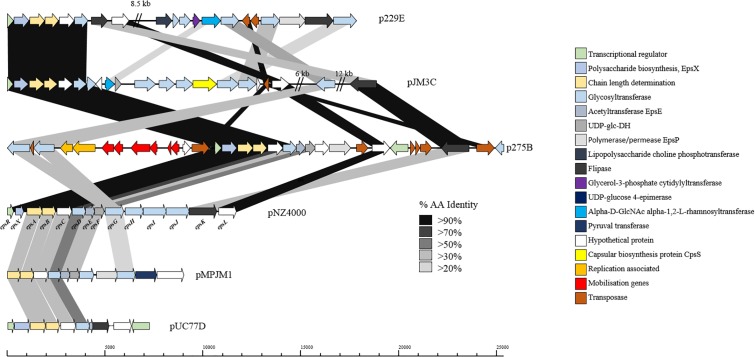
Linear BLAST map of the lactococcal EPS gene clusters. Linear BLAST map of *eps* gene clusters from (1) p229E, (2) pJM3C, (3) p275B, (4) pNZ4000, (5) pMPJM1, and (6) pUC77D. Arrow color indicates predicted product, while shaded region indicated percentage amino acid identity between BLAST hits. The highly conserved region of the gene cluster is apparent from EpsR to EpsD while the variable region is strain specific.

Exopolysaccharide production by *L*. *lactis* is a characteristic trait of strains isolated from viscous Scandinavian fermented milk products and is widely reported as a plasmid-encoded trait (Vedamuthu and Neville, 1986; Von Wright and Tynkkynen, 1987; Neve et al., 1988; Kranenburg et al., 1997). EPS production by *L*. *lactis* strains is of particular importance for functional foods, as the EPS produced by these strains is considered to be a food-grade additive that significantly contributes to properties such as mouth-feel and texture in fermented dairy products (Kleerebezem et al., 1999). The *L*. *lactis* EPS biosynthesis gene cluster (*eps*) contained on pNZ4000 has previously been characterized (Kranenburg et al., 1997) and consists of 14 genes, namely *epsRXABCDEFGHIJK*. Comparison of the *eps* gene cluster from pNZ4000 with all sequenced plasmids in the current dataset identified a further four plasmids harboring *eps* clusters, namely pUC77D, p229E, pJM3C, p275B, and pMPJM1 (Figure 6). In pNZ4000, EPS production is regulated by *epsRX*, EPS subunit polymerization and export is believed to be executed by the encoded products of *epsABIK*, while the proteins encoded by *epsDEFGH* are responsible for the biosynthesis of the EPS subunit (Kranenburg et al., 1997). Homology-based analysis with the five newly identified gene clusters shows that in all cases *epsRXABCD* are conserved (except in pMPJM1 where *epsR* is absent), while the remainder of the gene cluster in each case consists of variable genes. These *eps* gene clusters consist of a highly conserved region at the proximal end of the cluster and a variable distal region, which is not unlike other lactococcal polysaccharide biosynthesis clusters (Mahony et al., 2013; Ainsworth et al., 2014b; Mahony et al., 2015). The conserved *epsRX* genes are responsible for transcriptional regulation, the products of *epsAB* are required for EPS export, while the deduced proteins of *epsCD* are putative glycosyltransferases of which EpsD (priming glycosyltransferase) has previously been demonstrated to be essential for EPS subunit biosynthesis (Kranenburg et al., 1997). The variable region, *epsEFGHIJKLP* in pNZ4000, encodes predicted or proven functions, such as an acetyltransferase (*epsE*), glycosyltransferases (*epsGHIJ*) and a flippase (*epsK*), together representing the presumed enzymatic machinery responsible for EPS biosynthesis through the addition and export of sugar moieties.

In the case of p229E, the variable *eps* region is composed of CDSs predicted to encode products with functions are similar to the chromosomally located *cwps* gene cluster in strain 229. Plasmid pJM3C contains genes predicted to encode a rhamnosyltransferase, UDP-glucose dehydrogenase, capsular biosynthesis protein and five glycosyltransferases. The p275B variable region is heavily rearranged due to the presence of nine transposase-encoding genes. The megaplasmid pMPJM1 encodes a 9 Kbp predicted EPS region with well conserved functional synteny to that of pNZ4000, although with relatively low homology (Figure 6). Plasmid pUC77D appears to contain the shortest *eps* gene cluster of 7 Kbp due to the absence of *epsFGHIJ* genes. Further analysis of these plasmid-borne *eps* gene clusters revealed that in all cases *mob* elements are present, indicating that they may be mobilisable via conjugation. To assess if these plasmids have a common lineage, nucleotide homology based analysis was conducted utilizing BLASTN (Altschul et al., 1990). This analysis, however, did not identify significant homology or common hits between the plasmids outside of the conserved region of the EPS gene cluster. Phenotypic analysis of strains *L*. *lactis* 275, 229, JM1, JM3, and UC77 indicated a mucoid EPS phenotype in strains 275, 229, and JM3. While strains JM1 and UC77 did not show any EPS production which is probably attributed to the lack of the regulator epsR in strain JM1 and the absence of *epsFGHIJ* genes in UC77.

### Bacteriocins

Bacteriocins are a diverse group of ribosomally synthesized bacterial peptides, which when secreted inhibit growth of other bacteria by interfering with cell wall biosynthesis or disrupting membrane integrity (Dobson et al., 2012). The production of bacteriocins by lactococcal strains has been widely reported, including the strain *L*. *lactis* subsp. *cremoris* 9B4 which contains three separate bacteriocin operons, named lactococcins A, B, and M/N are located on one plasmid (Van Belkum et al., 1989, 1991). To investigate bacteriocin production in the lactococcal plasmidome, all the available strains were screened for bacteriocin production against an indicator strain *L*. *lactis* subsp. *cremoris* HP. In total six strains were found to produce clearly defined zones of inhibition, indicating bacteriocin production, namely *L*. *lactis* subsp. *lactis* IO-1, 184, UC06, UC08, UC11, and *L*. *lactis* subsp. *cremoris* 158. Analysis of the plasmid complement of each of these strains indicated that strains 158, UC06 and UC08 each possess a plasmid-borne bacteriocin gene cluster, while IO-1, 184, and UC11 contain a bacteriocin gene cluster of chromosomal origin. In each case, these were identified as lactococcin producers: p158A is predicted to be responsible for lactococcin A and B production, pUC08A for lactococcin A production, and pUC06C for lactococcin B biosynthesis. Lactococcin has a narrow spectrum of activity, targeting predominantly closely related lactococcal species (Geis et al., 1983) and, as such, is an important consideration when selecting strains for application in mixed starter cultures.

Sequence analysis of the remaining plasmids in the current study (for which strains were not available for phenotypic analysis) identified additional putative bacteriocin-encoding gene clusters (Table 3), which were found to be responsible for the production of lactococcin A or B, and in one case (pMRC01) for the lantibiotic lacticin 3147 (Table 3; Dougherty et al., 1998).

**Table 3:** Predicted plasmid-encoded antimicrobial peptides.

Plasmid	Bacteriocin	Activity detected
pBL1	Lactococcin 972	N/A^$^
pCIS7	Lactococcin A	Yes (Ainsworth et al., 2014a)
pMN5	LsbB bacteriocin	Yes (Kojic et al., 2006)
pMRC01	Lacticin 3147	Yes (Ryan et al., 1996)
SK11 plasmid 1	Lactococcin A	No
p158A	Lactococcin A and B	Yes
pUC08C	Lactococcin A	Yes
pUC06C	Lactococcin B	Yes
pA12-2	Lactococcin A	N/A

*^$^N/A, host strain unavailable to screen phenotypically.*

### Phage-Resistance Systems

Lactococcal strains typically possess a variety of phage defense mechanisms including superinfection exclusion systems (Sie) (encoded by integrated prophages) (Kelleher et al., 2018), clustered regularly interspaced short palindromic repeats (CRISPR), restriction-modification (R-M), and abortive infection (Abi) systems. Sie systems are a prophage-encoded defense mechanism (Mcgrath et al., 2002; Mahony et al., 2008) and have been reviewed extensively in these strains as part of an investigation into lactococcal prophages (Kelleher et al., 2018). CRISPR and CRISPR-associated (*cas*) genes specify an acquired adaptive immunity system against invading DNA in bacteria (Horvath and Barrangou, 2010). To date, only one such system has been characterized in *Lactococcus* on a conjugation-transmissible plasmid, pKLM, which encodes a novel type III CRISPR-Cas system (though it is unable to incorporate new spacers) (Millen et al., 2012). Analysis of plasmid sequences in this study did not detect any further instances of CRISPR systems in lactococci, suggesting CRISPR are not a widespread phenomenon in domesticated lactococci.

Restriction-modification systems are extremely diverse and widespread and are encoded by approximately 90% of all currently available bacterial and archaeal genomes (Roberts et al., 2003). R-M systems are frequently observed in the lactococcal plasmidome and some examples have previously been characterized including the Type II system LlaDCHI from pSRQ700 (Moineau et al., 1995) and LlaJI from pNP40 (O’driscoll et al., 2004). The current dataset holds nine apparently complete Type II systems on plasmids pCV56A, p275D, pJM1D, pUC08B, pUC11B, pNP40, pSRQ700, KLDS 4.0325 plasmid 5, and pAF22; along with multiple orphan methylases and solitary restriction endonucleases. The most commonly encountered R-M systems in lactococcal plasmids are Type I systems. These systems are often incomplete and represented by solitary specificity subunits (77 such orphan specificity subunit-encoding *hsdS* genes were identified in the current analysis). The high frequency of these systems in lactococcal plasmids is indicative of host adaptation as they predominantly act as a host defense mechanism against phage infection.

Abortive infection systems represent an abundant phage defense mechanism in *L*. *lactis* (Chopin et al., 2005) and are frequently plasmid-encoded (Mills et al., 2006). To date, 23 Abi systems have been identified in *L*. *lactis*, of which 21 are plasmid-encoded (Ainsworth et al., 2014c). Most are typically single gene systems, with the exception of three multigene systems, AbiE (Garvey et al., 1995), AbiR (Twomey et al., 2000), and AbiT (Bouchard et al., 2002). Analysis of the plasmids in this study identified eight Abi occurrences based on homology, namely AbiF, AbiC, AbiK, AbiQ, and two occurrences of the two component system AbiEi- AbiEii, alongside twelve predicted uncategorized Abi’s (Table 4), based on amino acid homology to unclassified Abi’s in the NCBI database. The relatively low observed abundance of Abi’s in such a large plasmid dataset is surprising and may be the result of the diversity of Abi’s with the possibility of as yet unidentified systems.

**Table 4:** Lactococcal Abi systems detected.

Similar to Abi system	Plasmid	Locus tag
AbiF	p158B	LL158_pB41
AbiF	pCIS8	UC509_RS11675
AbiF	pIL105	pIL105p7
AbiF	pNP40	pNP40_p16
AbiC	p275A	LL275_pA087
AbiEi-Eii	p275A	LL275_pA051-052
AbiEi-Eii	pNP40	pNP40_p19-20
AbiK	pSRQ800	pSRQ800_04
AbiQ	pCV56A	CVCAS_RS12180
AbiQ	pSRQ900	pSRQ900_04
Uncharacterized Abi^∗^	p158E	LL158_pE13
Uncharacterized Abi^∗^	pUC063B	LLUC063_pB07
Uncharacterized Abi^∗^	pCIS8	UC509_RS11625
Uncharacterized Abi^∗^	pUC08C	LLUC08_pC03
Uncharacterized Abi^∗^	pUC08C	LLUC08_pC04
Uncharacterized Abi^∗^	pUC08C	LLUC08_pC05
Uncharacterized Abi^∗^	p158E	LL158_pE13
Uncharacterized Abi^∗^	pUC063B	LLUC063_pB07
Uncharacterized Abi^∗^	pCIS8	UC509_RS11625
Uncharacterized Abi^∗^	pCIS5	UC509_RS12350
Uncharacterized Abi^∗^	pUC11D	LLUC11_pD04
Uncharacterized Abi^∗^	pCIS5	UC509_RS12350

*^∗^Uncharacterized Abi, based on amino acid homology to unclassified Abi’s in the NCBI database.*

## Discussion

The advent of NGS technologies has made genome sequencing much more accessible and has led to a dramatic rise in the number of available genome sequences. In the current study one such technology, SMRT sequencing was applied for the elucidation of 69 novel lactococcal plasmids. However, during the course of the current study some cautionary notes also emerged. These were predominantly related to smaller plasmids and plasmids with lower average consensus coverage, which could potentially be filtered out under standard assembly parameters. It was found that by performing the assembly using a reduced minimum coverage cut-off to 15-fold coverage detection of some of these plasmids was possible. In fact, in order to ensure detection of a given strain’s total plasmid complement we found it necessary to use a combined sequencing approach. This point is strongly supported by the elucidation of a further 14 plasmids from this dataset using an Illumina MiSeq approach which were completely absent from the SMRT assemblies.

The overview of plasmid replication systems presented shows that theta-type replication is the dominant way of replication used in *L*. *lactis*. These plasmids are usually viewed as being intrinsically more stable than RCR-type plasmids. However, a recent study of the dynamics of plasmid copy-number in *L*. *lactis* FM03-V1 demonstrated that the theta-type replicating plasmid (pLd10) was lost in a retentostat cultivation, while an RCR plasmid was maintained (Van Mastrigt et al., 2018c). During the course of that study, it was found that the reduced copy number of larger theta replicating plasmids increased the likelihood of the loss of these plasmids compared to smaller plasmids regardless of replication type (Van Mastrigt et al., 2018c), while the presence of the partition system (*parA* and *parB*) on these plasmids should also be considered as it has been shown to contribute to the stability and maintenance of large plasmids without selection (O’Driscoll et al., 2006). Interestingly, of the 16 plasmids not detected by SMRT sequencing in this study, five were theta replicating plasmids larger than 25 Kbp. This suggests that the lack of an amplification step during library preparation for SMRT sequencing may be a factor in detecting larger plasmids that may have a low copy number.

In the course of this study, the pan-plasmidome of *L*. *lactis* was calculated and found to be in a fluid state, making it likely that continued sequencing efforts would expand the diversity of this data set and lead to an increase in the identification of novel plasmid features. At present, the lactococcal plasmidome was found to consist of over ∼5000 Kbp of extra-chromosomal DNA encoding an arsenal of diverse features. Significantly, the current open plasmidome contributes the equivalent of 22.26% of the CDSs contained in the pan-genome of the *L*. *lactis* chromosomes that is in a closed state (Kelleher et al., 2017). BLAST-based analysis of these features identified 885 protein families, of which 413 represented unique families, evidence of the divergent nature of the plasmid sequences. There is, however, a skew in the data set toward the dairy niche, which has arisen due to a number of factors. Primarily, the majority of strains sequenced to date have been sequenced due to their commercial value in the production of fermented dairy products. The impact of these strains on the overall data set is further amplified as these strains generally carry a larger plasmid complement than their non-dairy counterparts (Kelleher et al., 2017), since many desirable dairy-associated traits are typically plasmid-encoded (e.g., *lac* operon). As such, these features account for a large proportion of the plasmidome. However, as efforts to isolate new starter cultures for the dairy industry continue (Cavanagh et al., 2015), screening of more diverse cultures, particularly from the plant niche, is expected to lead to increased novelty and diversity in the lactococcal plasmidome.

Megaplasmids have been found in LAB previously, in particular in members of the *Lactobacillus* genus (Muriana and Klaenhammer, 1987; Roussel et al., 1993; Claesson et al., 2006; Li et al., 2007; Fang et al., 2008). In the current study, sequencing efforts resulted in the identification of two examples of lactococcal megaplasmids (>100 Kbp), with pMPJM1 (193 Kbp) substantially surpassing the size of the previously largest sequenced plasmid in this taxon *L. lactis* S50 p7 (155 Kbp) (Kojic et al., 2005), and providing further diversity within the plasmidome. While megaplasmids are not expected to be essential for growth of their host, they can encode additional metabolic capabilities. The lactococcal megaplasmids were also examined for the presence of conjugation machinery. A novel gene cluster encoding a number of conjugation-related proteins located in pMPJM1 suggests that this plasmid is or has been involved in conjugal transfer. Further analysis of *mob* and *tra* genes across the plasmidome identified a number of genes predicted to encode proteins involved in conjugal transfer. The frequency (422 *mob/tra* genes across 190 plasmids) of these genes is indicative of the self-transmissible and/or mobilizable nature of lactococcal plasmids.

There has been limited research performed to date in the area of lactococcal gut adhesion as *L*. *lactis* is not commonly associated with the human gut. In this study, the lactococcal plasmidome was shown to contain potential gut adhesion factors, which may allow colonization and/or persistence in the gastrointestinal tract. This trait may offer opportunities for the application of *L. lactis* as a vector for vaccine and biomolecule delivery (Bermúdez-Humarán, 2009; Bermúdez-Humarán et al., 2013). Further technological properties of *L. lactis* were investigated including EPS production. Analysis of a large dataset of newly sequenced plasmids facilitated the identification and comparison of a number of novel EPS gene clusters. The major outcome of this work was the definition of “conserved” and “variable” regions within these EPS clusters. The conserved region encodes the transcriptional regulation, export and biosynthesis initiation machinery, while the variable region contains various genes that are predicted to encode glycosyltransferases, which are believed to be responsible for the production of a diverse set of EPS subunits, and thus a polysaccharide with a distinct composition and perhaps different technological properties.

Finally, phage-resistance mechanisms were assessed with particular emphasis on Abi systems. Abi systems confer defense against phage infection and are commonly found in lactococcal strains where they are frequently plasmid-encoded (Mills et al., 2006). Analysis of the plasmid sequences identified 22 plasmid-encoded Abi systems, while further analysis also identified frequent occurrences of these systems within the lactococcal chromosomes (Chopin et al., 2005). The presence of these systems and a range of R-M systems is evidence for the adaptation of these strains toward phage-resistance.

Discovery of the first lactococcal megaplasmids along with a host of novel features is evidence that the diversity of the lactococcal plasmidome represents a significant amount of unexploited genetic diversity, and suggests that continued future sequencing efforts and subsequent functional analysis will increase the observed diversity carried by these elements, potentially leading to new avenues of research, and applications. The current plasmidome contributes the equivalent of 22.26% of the CDSs contained in the pan-genome of the L. lactis chromosomes demonstrating its significant value to this taxon. The importance of which has been built on a long history of use in food fermentations, particularly in the dairy industry. The fact that both the opp and lac operons which have led to this adaptation remain largely plasmid encoded only further demonstrates the fundamental importance of the lactococcal plasmidome in terms of the evolution, adaptation, and application of lactococci.

## Data Availability

The datasets generated for this study can be found in NCBI Genbank, CP034577, CP034578, CP034579, CP034580, CP034581, CP034582, CP034583, CP034584, CP034585, and CP034586.

## Author Contributions

PK carried out the data analysis with FB. PK performed the experiments. DS and JM provided materials and strains. PK, JM, and DS wrote the manuscript. All authors read and approved the final manuscript.

## Conflict of Interest Statement

The authors declare that the research was conducted in the absence of any commercial or financial relationships that could be construed as a potential conflict of interest.

## Footnotes

1 http://compbio.ornl.gov/prodigal/

2 https://www.ncbi.nlm.nih.gov/

3 http://www.sanger.ac.uk/science/tools/artemis

4 http://www.uniprot.org/

5 http://www.tm4.org/mev.html

## Abbreviations

BLAST: basic local alignment search tool
CDS: coding sequence
HCL: hierarchical clustering
MCL: Markov clustering algorithm
NGS: next generation sequencing
ORF: open reading frame
PFGE: pulse field gel electrophoresis
qPCR: quantitative polymerase chain reaction
R-M: restriction modification
SMRT: single molecule real time sequencing.
